# The correlation of gait and muscle activation characteristics with locomotion dysfunction grade in elderly individuals

**DOI:** 10.3389/fbioe.2024.1372757

**Published:** 2024-08-05

**Authors:** Wen Liu, Jinzhu Bai

**Affiliations:** ^1^ Rehabilitation Medicine Center, The Second Affiliated Hospital and Yuying Children’s Hospital, Wenzhou Medical University, Wenzhou, China; ^2^ China Rehabilitation Research Center, Department of Spine and Spinal Cord Surgery, Beijing Boai Hospital, Beijing, China; ^3^ School of Rehabilitation Medicine, Capital Medical University, Beijing, China

**Keywords:** elderly, motor dysfunction, gait analysis, sEMG, LDG scale

## Abstract

**Objective:**

To investigate the differences and regularity of gait and muscle activation characteristics parameters in the Locomotion Dysfunction Grade (LDG) scale assessment in elderly individuals, and analyse the correlation between objective parameters and scale grading. Thus, to propose a novel detection mode for elderly individuals, which combined the LDG scale with objective detection. It can not only provide quantitative data for intelligent evaluation and rehabilitation, but also provided more accurate reference for the classification of care levels in elderly care policies.

**Methods:**

Elderly individuals (n = 159) who underwent gait analysis and sEMG at the Chinese Rehabilitation Research Center from January 2019 to September 2023 were included. According to the LDG scale, the elderly individuals were divided into four groups, namely, the LDG4, LDG5, LDG6 groups and the healthy control group. Four indicators, namely, spatiotemporal, kinematic, dynamic gait parameters and muscle activation characteristics data, were collected. Changes in these characteristics of elderly individuals with lower extremity motor dysfunction were evaluated and analysed statistically.

**Results:**

The spatiotemporal gait parameters were significantly lower in the LDG4, LDG5, LDG6 groups than in the healthy control group. The double support phase was positively correlated with the LDG, while the swing phase, step length and velocity were negatively correlated (*P* < 0.05). The movement angles of both hips, knees and ankles were significantly limited and negatively correlated with the LDG (*P* < 0.05). Compared with those in the healthy control group, the centre of pressure (COP) path length were greater, and the average COP velocity was significantly lower (*P* < 0.05) in the LDG4, LDG5, LDG6 groups. The regularity of muscle activation clearly changed. The root mean square of the gastrocnemius medialis was positively correlated with LDG (*P* < 0.05), while the tibialis anterior showed no regularity.

**Conclusion:**

As the LDG increased, the differences in spatiotemporal, kinematic and dynamic gait parameters between elderly individuals with motor dysfunction and the healthy individuals gradually increased. The muscle activation characteristics parameters showed an abnormal activation pattern. These parameters were correlated with the LDG, providing a more comprehensive and objective assessment of lower extremity motor function in elderly individuals, improve assessment accuracy, and help accurate rehabilitation.

## 1 Introduction

In the elderly individuals, due to aging or trauma, strength and motor ability decline, and joint rigidity and range of motion decrease, which leads to mobility disorders (Boyer et al., 2017; Jung et al., 2023). Moreover, the body’s ability to coordinate and balance, as well as the ability to judge and respond to dangerous situations, were also reduced (Song and Geyer, 2018). A delay in assessment and intervention can cause motor disability (Harridge and Lazarus, 2017). Iwaya et al. (Iwaya et al., 2017) reported a study on 711 elderly individuals and the results suggested that the Locomotion Dysfunction Grade (LDG) scale can track the progression of motor dysfunction and assess the effect of intervention. It can be easily implemented in a clinical setting, for example, by assessing the extent of a condition and identifying people who need medical or nursing support while also monitoring changes in functional status. LDG scale has good acceptable in the diagnosis of motor dysfunction and been proposed by the Japanese Long Term Care Insurance System (Kaigo Hoken) (Seichi et al., 2012; Iwaya et al., 2017). However, due to the elderly own confounding factors, such as physiology, psychology, cognition and behaviour, the assessment results may be subjective and rough. Moreover, differences in individuals and ages can lead to differences in motor ability and movement disorders; therefore, a single scale is insufficient for a comprehensive and accurate assessment of motor function (Riahi et al., 2020). Therefore, it is highly important to find more objective, sensitive and specific assessment tools for screening and assessing extremity motor dysfunction in elderly individuals.

Gait analysis based on inertial measurement units (IMUs) is considered the vital means for assessing lower extremity motor function (Zago et al., 2018). It follows the basic principles of biomechanics, human anatomy and physiology to detect and record body and joint movement, plantar pressure distribution and other data during a specific walking phase (Huang et al., 2022). Researches had shown that it have excellent effectiveness and reliability in determining walking parameters (Kobsar et al., 2020). It can simplify and improve the efficiency of gait data assessment and interpretation (Mazzetta et al., 2019). The prediction accuracy of fall risk and walking ability was significantly improved in elderly individuals (Tahir et al., 2022). As an important method for muscle function assessment, surface electromyography (sEMG) can provide a non-invasive and dynamic neuromuscular function status detection. By measuring and recording the sum of action potentials of motor units under electrodes, it reflects changes in muscle load or muscle recruitment. When voluntary muscle contractions are detected, sEMG signals could tell us the muscle activity of the elderly individuals during gait, identifying the abnormal activation patterns of muscles, which may affect walking function (Martin and Acosta-Sojo, 2020). It shows good potential and value in evaluating motor function status of elderly individuals with osteoarthritis, Parkinson’s disease, hemiplegia, etc (de Oliveira et al., 2019; Booij et al., 2020; Daniels and Knight, 2021).

Based on the improved Ashworth score, some scholars had objectively evaluated motor parameters and muscle activation levels, using IMU and sEMG to capture motion and electromyographic signals (Ang et al., 2018). However, there is no clear evaluation standard for motor dysfunction in the elderly individuals. Based on the LDG scale, gait analysis and sEMG was performed in this study to evaluate the regularity of gait parameters and muscle activation in elderly individuals with lower extremity motor dysfunction. At present, there were few studies on the correlation between LDG scale and objective detection. By identifying the correlation of LDG scale grading with gait and muscle activation parameters, the subjective evaluation bias of the scale can be reduced and the accuracy of the evaluation can be improved. At the same time, the combination of scale and objective detection as a new evaluation method can provide evidence for intelligent evaluation and rehabilitation, and digital medicine, promoting accurate rehabilitation. This also provides more accurate reference for the classification of care levels in elderly care policies.

## 2 Methods

### 2.1 Participants

This study was approved by the ethics board of the Chinese Rehabilitation Research Center (register No. 2023-083-01), and 159 elderly individuals who underwent gait analysis and sEMG at the Chinese Rehabilitation Research Center were included from January 2019 to September 2023. The inclusion criteria for elderly individuals were as follows: (a) aged 60-80 years; (b) no contraindication for neuroelectrophysiological examination; and (c) had at least two complete gait cycles within the effective camera range. The exclusion criteria for elderly individuals were as follows: (a) had a history of lower extremity injury or deformity that seriously affected walking function; (b) had cognitive impairment and refused to cooperate; (c) had received treatments for acute trauma; (d) had a history of lower extremity and/or spinal fracture within the past 6 months; and (e) lacked gait and sEMG data (5, 6).

### 2.2 LDG scale

According to the criteria, the elderly individuals were divided into four groups: the LDG4, LDG5, and LDG6 groups and the healthy control group. The specific classification criteria are as follows: LDG4 group is a mild activities of daily living (ADL) dysfunction that can walk independently without assistance; LDG5 group is a moderate ADL dysfunction and can walk independently without assistance; LDG6 group is a moderate or severe ADL dysfunction that can walk with support (Seichi et al., 2012; Iwaya et al., 2017).

### 2.3 Experimental protocol and acquisition system

MyoMotion three-dimensional motion acquisition and analysis system (Noraxon, USA, sampling frequency 100 Hz) and MyoPressure plantar pressure acquisition and analysis system (Noraxon, USA, sampling frequency 120 Hz) were used for gait detection. A wireless sEMG tester (Noraxon, USA, sampling frequency 1,500 Hz)was used to synchronize the signals, and MATLAB (MathWorks, Natick, USA) was used to process the signals (Hallal et al., 2013).

**FIGURE 1:**
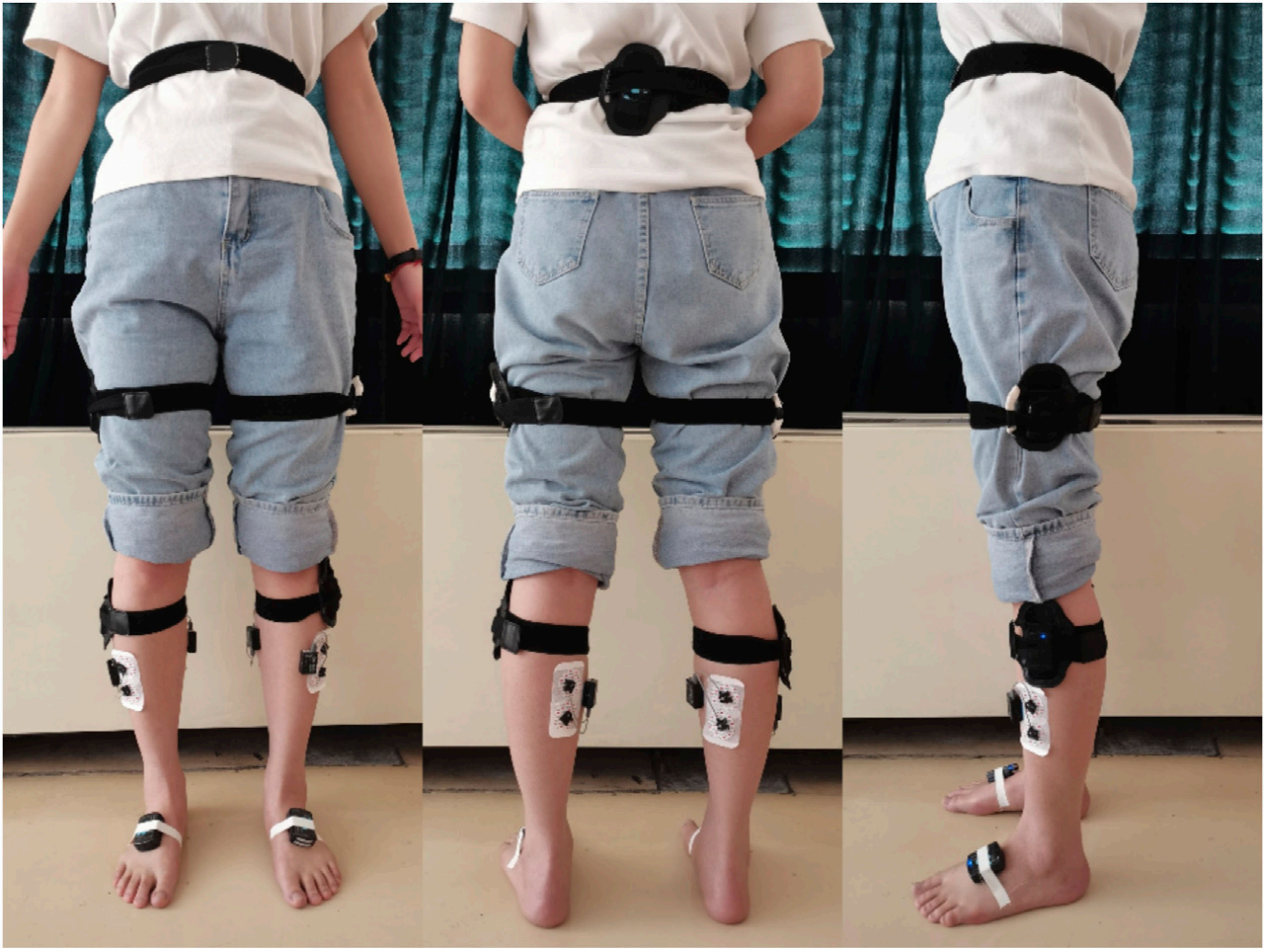
Location of gait sensor and sEMG sensor. The pelvis sensor was fixed to the sacrum, and the thighs sensors were fixed to middle lateral thigh that halfway between the hip and knee joints. The shanks sensors were fixed to hard surface of tibial bone that below the knee and above the thickest part of the calf. The feet sensors were fixed to dorsal feet that under the tongue of the participant’s shoe, approximately over the distal end of the third and fourth metatarsal bones.

Before starting the test, explain the process and precautions to the elderly individuals, to ensure that they fully understand and cooperate. First, the skin area under the electrodes was shaved, cleaned with ethyl alcohol, abraded gently with fine sandpaper. Following the European Recommendations for Surface Electromyography (Hermens et al., 1999) and the guidelines as stated by the SENIAM Project (Surface Electromyography for the Non-Invasive Assessment of Muscles), the sEMG electrodes were placed on the tibialis anterior (TA) and the gastrocnemius medialis (GM) (Hermens and Freriks, 2019; SENIAM et al., 2019), along the direction of the muscle fibres. Seven Noraxon MyoMotion IMU lower extremity sets were placed on the participants while standing in the anatomical position. The pelvis, thighs, shanks, and feet IMUs were equipped on the sacrum, lateral femur, lateral tibia and dorsal feet of the elderly individuals (Niswander et al., 2020; Rekant et al., 2022). Detailed anatomical locations were shown in Figure 1. The sensors were wrapped in elastic wrap to prevent a sensor from moving from its original place (Rantalainen et al., 2020).

The IMU-based body model for calculating joint angle followed the recommendations of the International Society of Biomechanics (ISB) (Wu et al., 2002). The kinematics data were derived from relationships between coordinate systems (*x*-axis: pointing towards the top of the IMU along its length, *y*-axis: pointing to the left of the IMU, *z*-axis: pointing outwards perpendicular to IMU surface). MATLAB used IMU’s vertical foot accelerometer data to identify heel strikes and extract temporal features of gait. Then trials were parsed into gait cycles using heel strike timings and resampled to data points per gait cycle (Rekant et al., 2022). The motion angles output of the hip, knee and ankle joints were automatically calculated by MATLAB.

The camera was fixed on the side of the middle area of the walking path and were synchronized with the MyoMotion system. Before initiating the study, the MyoMotion system was calibrated according to the manufacturer’s guidelines. The session started with familiarization to walking on the plantar pressure board while selecting the natural speed. For each participant, a calibration trial was performed before the test. During the calibration, the participant was instructed to stand in the anatomical position for 10 s (Oliveira et al., 2023). Then walk took place on the plantar pressure board back and forth twice with a preferred speed to obtain average result. The walking length was 6 m, and the plantar pressure board was centered to ensure that it reflects the natural state of walking. All the measurements were performed by rehabilitation specialists, and the room was quiet during the measurements. The safety of all elderly individuals in the study was guaranteed.

### 2.4 Outcome measures

Basic information such as sex, age, height and weight was recorded for the study participants. Four types of indicators were collected and analyzed: gait spatiotemporal parameters, including velocity, step length, cadence, stride length, walking time, step width, walking cycle, etc.; kinematic parameters, including the range of motion of bilateral hip, knee, and ankle joints and 95% confidence ellipse region, centre of pressure (COP) path length, COP average velocity; dynamic parameters, including pressure peak, pressure start time ratio, and muscle activation parameters, including average power frequency (MPF) and median frequency (MF) of the TA and the GM at different walking cycles.

### 2.5 Statistical analysis

All the statistical analyses were performed with SPSS (version 20.0, IBM Corp., Armonk, NY, USA). Gait and sEMG data were analyzed using MATLAB (Molinari et al., 2006; Merletti et al., 2009), and standardized parameters were obtained after noise reduction. The original sEMG data was processed by full wave rectification, smooth filtering and RMS processing, and the window constant was set to 200 ms. All RMS value in each gait cycle were calculated respectively, and the average RMS was obtained after normalization processing. Then the distribution diagram of RMS in the gait cycle was obtained, so as to get the average standing RMS and swinging RMS. The detailed processing methods and operational interfaces could be found in Supplementary Appendix S1.

Spearman correlation analysis was performed using SPSS to analyze the correlation between gait or muscle activation parameters and scale grade. The normally distributed data are reported as the means and standard deviations (SDs), and the intragroup and intergroup differences were evaluated by one-way ANOVA. Skewed data are reported as the medians and interquartile distances, and the intragroup and intergroup differences were evaluated by rank-sum tests. Tukey’s test and Dunn-Bonferroni test were used for *post hoc* multiple comparisons (Szpala et al., 2022). *P* < 0.05 was considered statistically significant.

## 3 Results

In total, 159 elderly individuals were included according to the inclusion criteria: 45 in the LDG4 group, 42 in the LDG5 group, 38 in the LDG6 group and 34 in the healthy control group from the Chinese Rehabilitation Research Center. There was no statistically significant difference in the baseline data among the four groups (*P* > 0.05) (Table 1). Spearman analysis indicated that the spatiotemporal parameters, kinematic parameters, dynamic parameters and muscle activation parameters were correlated with the LDG (Table 2).

**TABLE 1:** Comparison of general data between LDG 4, 5, 6 groups and healthy control group.

	LDG6 (a)	LDG5 (b)	LDG4 (c)	Healthy controls (d)	X^2^/F	Adjusted P
Numbers	45	42	38	34	—	
Gender (M: F)	33:12	28:14	30:8	8:26	—	
Age (years)#	67.00 (7)	64.5 (6)	66.00 (7)	65.00 (9)	3.138	0.371
Height (cm)	166.311 ± 7.292	168.571 ± 7.302^d^	168.921 ± 6.839^d^	162.147 ± 7.233	6.767	0.001*
Weight (kg)	69.957 ± 10.755	67.279 ± 8.194	71.316 ± 11.471	65.868 ± 13.142	1.870	0.138
BMI	25.232 ± 3.126	23.646 ± 2.169	24.907 ± 3.0409	24.968 ± 4.152	2.040	0.112

Note: #:The skewed distribution data are reported as the median and interquartile distances.

M:F: Male:female. LDG: Locomotion Dysfunction Grade. BMI: body mass index.

*Indicates P< 0.05, which was considered to indicate statistical significance.

After multiple comparisons using Tukey’s test and Dunn-Bonferroni test, d indicates P< 0.05 when compared to group (d).

**TABLE 2:** Correlation analysis of spatiotemporal, kinematic and dynamic gait parameters and muscle activation parameters.

		95%CI	Spearman	P			95%CI	Spearman	P
stance phase (%)	Left	(-0.564, −0.321)	−0.450	0.000**	hip flexion	Stance left	(0.354, 0.615)	0.496	0.000**
	Right	(-0.593, −0.332)	−0.472	0.000**		Stance right	(0.300, 0.561)	0.440	0.000**
load response (%)	Left	(-0.579, −0.333)	−0.461	0.000**		Swing left	(0.295, 0.565)	0.442	0.000**
	Right	(-0.587, −0.309)	−0.457	0.000**		Swing right	(0.305, 0.577)	0.449	0.000**
mid stance (%)	left	(0.298, 0.558)	0.433	0.000**	hip abduction	Stance left	(-0.008, 0.297)	0.144	0.071
	right	(0.333, 0.599)	0.472	0.000**		Stance right	(0.003, 0.294)	0.153	0.054
pre-swing (%)	left	(-0.621, −0.360)	−0.502	0.000**		Swing left	(0.183, 0.458)	0.321	0.000**
	right	(-0.615, −0.386)	−0.507	0.000**		Swing right	(0.189,0.465)	0.334	0.000**
swing phase (%)	left	(0.319, 0.564)	0.449	0.000**	hip rotation	Stance left	(-0.158, 0.165)	0.001	0.986
	right	(0.332, 0.593)	0.472	0.000**		Stance right	(-0.067, 0.251)	0.098	0.218
double stance (%)		(-0.639, −0.397)	−0.524	0.000**		Swing left	(0.113, 0.397)	0.257	0.001**
foot rotation	left	(-0.323, −0.008)	−0.173	0.030*		Swing right	(-0.013, 0.284)	0.132	0.098
	right	(-0.196, 0.112)	−0.046	0.567	knee flexion	Stance left	(0.111, 0.393)	0.249	0.002**
step length (cm)	left	(0.473, 0.683)	0.587	0.000**		Stance right	(0.129, 0.436)	0.289	0.000**
	right	(0.435, 0.667)	0.561	0.000**		Swing left	(0.415, 0.646)	0.535	0.000**
stride length (cm)		(0.487, 0.688)	0.595	0.000**		Swing right	(0.395, 0.647)	0.535	0.000**
step width (cm)		(-0.395, −0.096)	−0.260	0.001**	ankle dorsiflexion	Stance left	(0.172, 0.468)	0.330	0.000**
velocity (km/h)		(0.514, 0.716)	0.622	0.000**		Stance right	(0.203, 0.493)	0.359	0.000**
Cadence (steeps/min)		(0.314, 0.560)	0.442	0.000**		Swing left	(0.198, 0.497)	0.355	0.000*
time(s)#	left	(-0.567, −0.327)	−0.452	0.000**		Swing right	(0.173, 0.479)	0.331	0.000*
	right	(-0.492, −0.219)	−0.365	0.000**	ankle inversion	Stance left	(-0003, 0.293)	0.149	0.061
						Stance right	(0.078, 0.390)	0.241	0.002**
						Swing left	(0.173, 0.450)	0.313	0.000**
95% confidence ellipse		(-0.269, 0.051)	−0.121	0.130		Swing right	(0.220, 0.509)	0.367	0.000**
COP path length		(-0.685, −0.466)	−0.583	0.000**	ankle abduction	Stance left	(0.204, 0.465)	0.328	0.000**
average COP velocity		(0.356, 0.584)	0.477	0.000**		Stance right	(-0.077, 0.233)	0.081	0.309
landing style	toe landing	(-0.217, 0.133)	−0.029	0.714		Swing left	(0.275, 0.544)	0.413	0.000**
	foot landing	(-0.133, 0.217)	0.029	0.714		Swing right	(0.206, 0.503)	0.357	0.000**
peak pressure	the heel left	(-0.207, 0.106)	−0.052	0.512	the ratio of pressure onset time#	the heel left	(-0.301, 0.004)	−0.148	0.062
	the heel right#	(-0.276, 0.037)	−0.125	0.116		the heel right	(-0.203, 0.080)	−0.063	0.433
	middle foot left	(-0.022, 0.293)	0.139	0.079		middle foot left	(0.133, 0.423)	0.275	0.000**
	middle right#	(-0.082, 0.242)	0.079	0.323		middle foot right	(0.252, 0.523)	0.384	0.000**
	anterior foot left	(0.335, 0.579)	0.467	0.000**		anterior foot left	(0.236, 0.503)	0.368	0.000**
	anterior foot right	(0.330, 0.563)	0.448	0.000**		anterior foot right	(0.312, 0.566)	0.441	0.000**
TA	Left Standing RMS(μV)	(-0.018, 0.307)	0.150	0.060	GM	Left Standing RMS(μV)	(0.103, 0.401)	0.255	0.001**
	Left Swing (μV)	(0.042, 0.343)	0.190	0.016*		Left Swing RMS(μV)	(0.092, 0.397)	0.249	0.002**
	Left MPF	(-0.017, 0.301)	0.144	0.071		Left MPF	(-0.240, 0.080)	−0.083	0.297
	Left MF	(-0.034, 0.274)	0.122	0.125		Left MF	(-0.207, 0.108)	−0.051	0.525
	Right Standing RMS(μV)	(-0.131, 0.175)	0.020	0.807		Right Standing (μV)	(0.059, 0.364)	0.209	0.008**
	Right Swing RMS(μV)	(-0.059, 0.248)	0.096	0.228		Right Swing RMS(μV)	(-0.013, 0.316)	0.150	0.059
	Right MPF	(-0.041, 0.302)	0.123	0.124		Right MPF	(-0.102, 0.217)	0.053	0.506
	Right MF	(-0.024, 0.305)	0.131	0.100		Right MF	(-0.130, 0.193)	0.033	0.676

Note: 95% CI: 95% confidence interval. COP: center of pressure. RMS: root mean square. TA: tibial anterior, GM: gastrocnemius medialis. MPF: average power frequency; MF: median frequency.

*Indicates *P* < 0.05, which was considered to indicate statistical significance; ** indicates *P* < 0.01, which was considered to indicate statistical significance.

### 3.1 Gait spatiotemporal parameters

Compared with those in the healthy control group, the step velocity and cadence in the LDG4, 5, 6 groups were lower, the step length and stride length were shorter, the walking time was significantly greater, and the step width was significantly greater (*P* < 0.05). Compared with those in the LDG4 group, the four parameters bilateral step length, stride length, velocity and cadence in the LDG6 group showed a more significant downward trend (P< 0.05). Similarly, compared with those in the LDG5 group, the velocity and cadence in the LDG6 group also showed a gradual downward trend (*P* < 0.05) (Supplementary Figure S1). There was no significant difference in foot rotation between the two sides (*P* > 0.05). During the gait cycle, the bilateral stance phase, load response, preswing phase and double stance phase significantly increased (*P* < 0.05), while the bilateral middle stance phase and swing phase significantly decreased in the four groups (*P* < 0.05) (Table 3).

**TABLE 3:** Comparison of spatiotemporal gait parameters between the LDG 4, 5, 6 groups and healthy control group.

		LDG6 (a)	LDG5 (b)	LDG4 (c)	Healthy controls (d)	X^2^/F	Adjusted P
stance phase (%)#	left	75.400 (12.300)^d^	73.700 (9.380)^d^	71.600 (12.550)^d^	67.500 (2.680)	38.530	0.001*
	right	75.600 (17.800)^d^	73.150 (11.980)^d^	70.400 (11.550)^d^	66.200 (3.280)	40.642	0.002*
load response (%)#	left	23.400 (12.000)^d^	23.200 (12.400)^d^	20.250 (9.050)	17.300 (4.550)	38.057	0.000*
	right	26.200 (13.550)^d^	23.550 (9.300)^d^	23.100 (13.300)^d^	17.050 (3.570)	39.344	0.001*
middle stance (%)#	left	23.900 (17.000)^d^	25.900 (14.650)^d^	29.050 (12.550)	33.300 (4.680)	32.851	0.000*
	right	25.300 (12.800)^d^	25.750 (9.450)^d^	27.300 (14.430)^d^	33.050 (4.350)	41.663	0.000*
pre-swing (%)#	left	25.200 (15.000)^d^	23.150 (9.650)^d^	21.650 (12.780)^d^	16.600 (2.570)	45.567	0.001*
	right	22.800 (12.650)^d^	23.200 (10.050)^d^	20.550 (8.280)^d^	16.000 (2.600)	50.149	0.000*
swing phase (%)#	left	24.600 (12.300)^d^	26.150 (9.380)^d^	28.400 (12.550)^d^	32.500 (2.670)	38.605	0.000*
	right	24.400 (17.800)^d^	26.850 (11.970)^d^	29.600 (11.550)^d^	33.800 (3.280)	40.642	0.000*
double stance (%)#		51.100 (26.450)^d^	47.250 (19.950)^d^	41.600 (22.130)^d^	33.850 (4.480)	48.835	0.000*
foot rotation	left	11.284 ± 6.899	10.129 ± 5.500	11.095 ± 6.454	8.209 ± 5.021	1.968	0.121
	right	13.527 ± 6.351	14.264 ± 6.886	14.524 ± 6.627	11.932 ± 6.067	1.148	0.332
step length (cm)	left	24.800 ± 9.104^c,d^	29.500 ± 9.789^d^	34.211 ± 12.779^d^	46.382 ± 7.114	32.937	0.000*
	right	26.111 ± 11.185^c,d^	30.571 ± 10.229^d^	34.737 ± 13.005^d^	47.235 ± 6.135	28.518	0.000*
stride length (cm)		50.978 ± 17.523^c,d^	59.976 ± 19.022^d^	68.947 ± 25.137^d^	93.441 ± 12.324	34.533	0.000*
step width (cm)		14.622 ± 3.875^d^	15.429 ± 3.351^d^	14.290 ± 3.601^d^	11.618 ± 3.330	7.809	0.000*
velocity (km/h)		1.013 ± 0.553^c,d^	1.381 ± 0.610^d^	1.616 ± 0.795^d^	2.574 ± 0.517	41.997	0.000*
cadence (steeps/min)		63.511 ± 21.557^b,c,d^	74.643 ± 21.380^d^	74.658 ± 17.535^d^	91.118 ± 11.020	14.797	0.000*
time(s)#	left	0.910 (0.730)^d^	0.760 (0.350)^d^	0.765 (0.270)^d^	0.650 (0.130)	37.076	0.002*
	right	0.870 (0.560)^d^	0.790 (0.380)^d^	0.750 (0.320)^d^	0.660 (0.170)	25.580	0.006*

Note: #:The skewed distribution data are reported as the median and interquartile distances.

M:F: Male:female. LDG: Locomotion Dysfunction Grade. BMI: body mass index.

*Indicates *P* < 0.05, which was considered to indicate statistical significance.

After multiple comparisons using Tukey’s test and Dunn-Bonferroni test.

^a-d^ indicates *P* < 0.05 when compared to group (a)- (d).

### 3.2 Gait kinematic parameters

Compared with those in the healthy control group, the joint motion in the LDG4, 5, 6 groups was significant different, and the overall angle showed a downward trend (*P* < 0.05). In the standing phase, bilateral hip flexion, knee flexion and the ankle dorsiflexion angle decreased in the LDG4, 5, 6 groups. Additionally the right ankle inversion and left ankle abduction angle decreased. There were no significant differences in the abduction or rotation angle of the bilateral hip joint or in the left ankle inversion or right ankle abduction angle (*P* > 0.05). In the swing phase, bilateral hip flexion, hip abduction, knee flexion, ankle dorsoextension, ankle inversion and the ankle abduction angle were slightly lower in the LDG4, 5, 6 groups than in the control group, and the left hip rotation angle was decreased (*P* < 0.05). Moreover, there was no significant difference in the right hip rotation angle (*p* > 0.05) (Table 4).

**TABLE 4:** Comparison of gait kinematic characteristics between the LDG 4, 5, 6 groups and healthy control group.

			LDG6 (a)	LDG5 (b)	LDG4 (c)	Healthy controls (d)	X^2^/F	Adjusted P
hip flexion	stance	left	22.490 ± 7.135^d^	25.557 ± 6.076^d^	26.192 ± 8.190^d^	33.703 ± 4.221	19.635	0.000*
		right	23.036 ± 7.014^d^	23.926 ± 5.927^d^	25.532 ± 7.376^d^	32.777 ± 5.027	17.529	0.000*
	swing	left	13.225 ± 7.029^d^	14.909 ± 6.211^d^	15.773 ± 7.703^d^	24.074 ± 5.198^d^	19.312	0.000*
		right	12.530 ± 6.971^d^	13.288 ± 5.862^d^	14.500 ± 6.458^d^	22.379 ± 5.103^d^	19.484	0.000*
hip abduction	stance	left	7.609 ± 3.402	7.804 ± 2.892	8.091 ± 3.309	8.569 ± 2.923	0.664	0.576
		right#	7.340 (3.720)	7.475 (4.540)	7.020 (3.360)	8.775 (3.380)	7.787	0.051
	swing#	left	3.140 (3.100)^d^	4.610 (4.600)	4.010 (4.560)	6.230 (4.530)	18.441	0.000*
		right	3.470 (3.600)^d^	3.540 (2.890)^d^	4.245 (4.760)	7.035 (4.780)	22.276	0.000*
hip rotation	stance	left	10.802 ± 4.501	9.662 ± 3.303	9.818 ± 4.416	11.028 ± 4.035	1.099	0.351
		right	10.104 ± 4.186	9.628 ± 3.356	10.472 ± 4.654	11.642 ± 5.148	1.449	0.231
	swing	left	7.535 ± 4.766	7.315 ± 4.560^d^	8.428 ± 4.101	10.008 ± 3.468	2.984	0.045*
		right	7.979 ± 4.535	7.635 ± 4.332	9.071 ± 5.124	9.984 ± 5.913	1.768	0.156
knee flexion	stance	left	32.420 ± 9.016^d^	36.033 ± 8.487	36.895 ± 8.283	39.679 ± 10.030	4.452	0.003*
		right	32.311 ± 10.061^d^	34.466 ± 9.010^d^	36.313 ± 9.218	41.118 ± 8.827	6.098	0.014*
	swing	left	29.027 ± 12.623^c,d^	35.522 ± 13.170^d^	37.171 ± 13.006^d^	51.135 ± 7.054	23.721	0.026*
		right	28.857 ± 12.274^c,d^	33.579 ± 12.658^d^	37.237 ± 14.601^d^	50.835 ± 8.401	22.496	0.032*
ankle dorsiflexion#	stance	left	17.300 (8.900)^d^	18.450 (8.180)^d^	18.600 (9.880)^d^	26.050 (8.520)	24.723	0.000*
		right	21.600 (8.500)^d^	25.500 (10.380)	24.350 (9.050)^d^	30.600 (13.350)	26.876	0.003*
	swing	left	7.720 (6.970)^d^	7.655 (5.620)^d^	8.435 (6.180)^d^	12.750 (7.010)	29.032	0.000*
		right	10.100 (5.440)^d^	11.650 (7.710)^d^	10.650 (5.880)^d^	16.450 (11.430)	23.347	0.005*
ankle inversion	stance	left	20.190 ± 9.767	20.328 ± 7.720	22.465 ± 11.931	22.671 ± 8.258	0.768	0.513
		right#	11.300 (9.870)^d^	12.900 (7.180)^d^	13.250 (9.630)	16.700 (9.500)	12.161	0.007*
	swing#	left	7.460 (4.700)^d^	9.665 (10.670)^d^	9.950 (7.430)	12.650 (9.900)	16.721	0.000*
		right	6.360 (5.230)^d^	8.040 (8.030)^d^	7.805 (4.310)^d^	14.200 (11.460)	30.832	0.000*
ankle abduction	stance	left#	8.440 (5.610)^d^	11.000 (8.890)^d^	10.900 (8.840)	13.450 (12.280)	19.172	0.000*
		right	10.724 ± 4.061	10.766 ± 3.702	11.724 ± 6.265	12.491 ± 5.437	1.133	0.337
	swing	left#	5.320 (5.170)^d^	5.325 (6.400)^d^	6.635 (4.910)^d^	10.150 (10.170)	32.958	0.000*
		right	6.220 ± 2.987^d^	6.078 ± 2.382^d^	7.847 ± 5.612	10.637 ± 4.711	9.574	0.000*
95% confidence ellipse			836084.756 ±157467.321	920328.095 ±175853.773^d^	903941.368 ±199719.939^d^	759807.147 ±181219.763	6.227	0.001*
COP path length#			2930.000 (1,356.000)^d^	2576.500 (747.000)^d^	2438.000 (650.000)^d^	2084.000 (193.000)	40.080	0.000*
average COP velocity			161.778 ±49.445^c,d^	191.698 ±60.740^d^	208.737 ±60.644^d^	251.559 ±61.234	16.130	0.011*
landing style#	toe landing		67.330 (5.090)	67.975 (4.090)	66.080 (6.450)	67.730 (9.990)	3.417	0.332
	foot landing		32.670 (5.090)	32.025 (4.090)	33.920 (6.450)	32.270 (9.990)	3.417	0.332

Note: #:The skewed distribution data are reported as the median and interquartile distances.

COP: center of pressure.

* Indicates P< 0.05, which was considered to indicate statistical significance.

After multiple comparisons using Tukey’s test and Dunn-Bonferroni test.

^a-d^ indicates P< 0.05 when compared to group (a)- (d).

Compared with those in the healthy control group, the 95% confidence ellipse and centre of pressure (COP) path length were greater, and the average COP velocity was significantly lower in the LDG4, 5, 6 groups (*p* < 0.05). In terms of landing style, no significant difference was found between toe landing and foot landing (*P* > 0.05) (Table 4; Supplementary Figure S2).

### 3.3 Gait dynamic parameters

**FIGURE 2:**
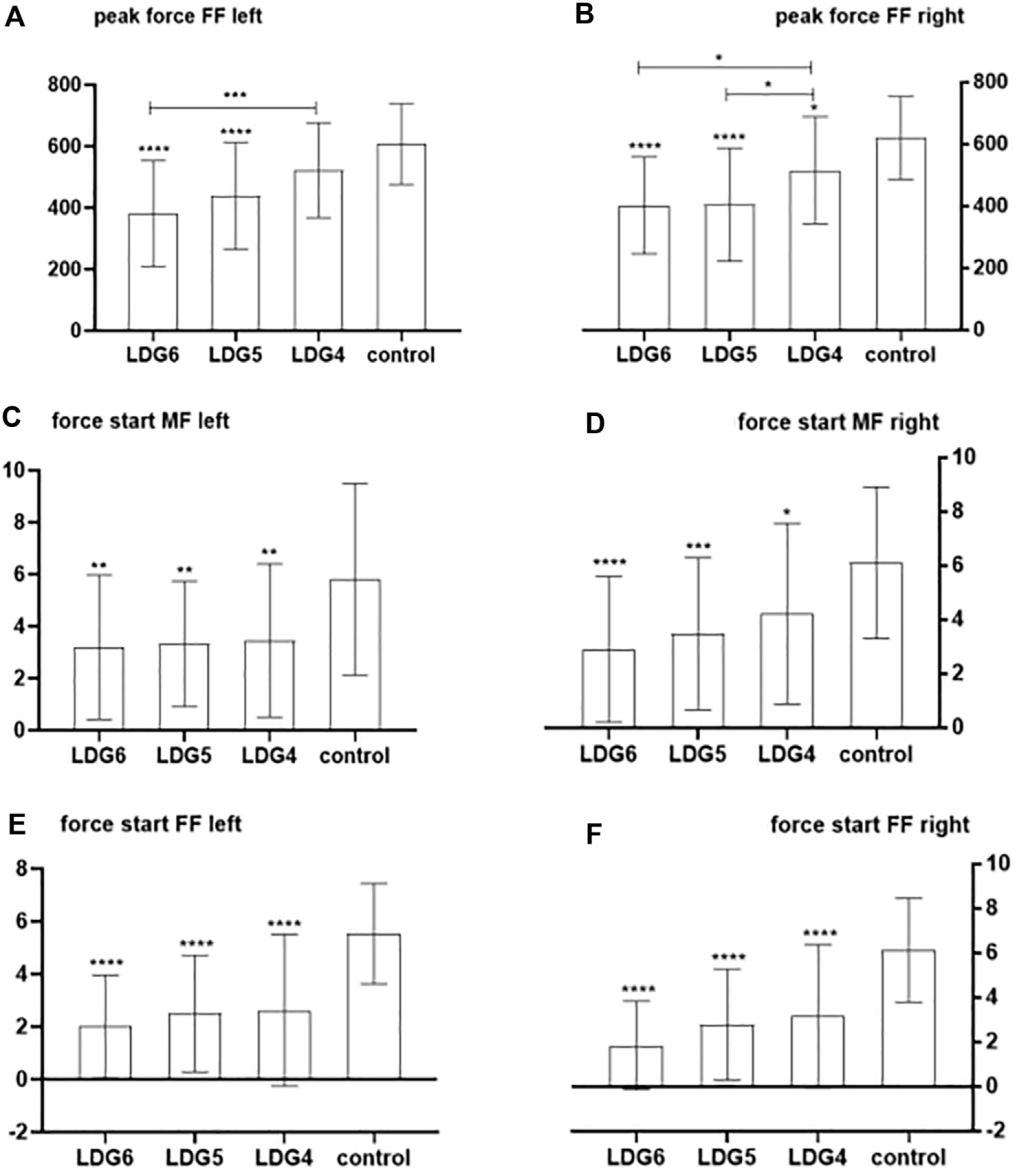
Comparison of the bilateral anterior foot and middle foot pressure peak and the ratio of bilateral anterior foot pressure onset time between the LDG4, 5, 6 groups and the healthy control group. Note: FF: anterior foot; MF:middle foot. **(A)** Peak pressure in the left anterior foot; **(B)** peak pressure in the right anterior foot; **(C)** peak pressure in the left middle foot; **(D)** peak pressure in the right middle foot; **(E)** ratio of pressure onset time in the left anterior foot; **(F)** ratio of pressure onset time in the right anterior foot.

The peak pressure and the ratio of pressure onset time were significantly different between the LDG4, 5, 6 groups and the healthy control group (*P* < 0.05). In terms of peak pressure, compared with that of healthy elderly individuals, the peak pressure in the bilateral anterior foot decreased step by step in the LDG4, 5, 6 groups, especially in the LDG6 group, which indicated that the feet were weak at the end of the standing phase. However, there was no significant difference in heel peak pressure among the four groups (*P* > 0.05). Moreover, there was a significant difference in the peak pressure in the left midfoot among the four groups (*P* < 0.05), but no regularity was observed. There was no significant difference in the peak pressure in the right midfoot (*P* > 0.05) (Figures 2A–D). Moreover, in the LDG4, 5, 6 groups, the bilateral pressure distribution was abnormal, and the pressure curve was not smooth, which suggested that the LDG4, 5, 6 groups had poor stability in the standing phase for both lower extremities (Supplementary Figure S3). In terms of the ratio of pressure onset time, compared with those in the healthy control group, the bilateral anterior foot and middle foot in the LDG4, 5, 6 groups showed a gradual downward trend (*P* < 0.05) (Table 5; Figures 2E, F).

**TABLE 5:** Comparison of gait dynamic parameters and muscle activation characteristics between the LDG 4, 5, 6 groups and healthy control group.

			LDG6 (a)	LDG5 (b)	LDG4 (c)	Healthy controls (d)	X^2^/F	Adjusted P
peak pressure	the heel	left	396.488± 103.386	401.502± 87.429	414.684± 100.713	376.294± 74.963	1.046	0.374
		right	414.487 ± 97.590	404.145± 96.913	414.716± 89.564	377.227± 84.969	1.299	0.277
	middle foot	left	153.980 ± 74.066	131.167± 66.820^d^	145.771± 63.855^d^	199.582± 106.935	4.900	0.025*
		right#	128.100 (72.600)	116.600 (103.530)	139.950 (112.750)	133.500 (107.650)	1.189	0.756
	anterior foot	left	380.189 ± 172.490^c,d^	437.783± 173.736^d^	522.071± 154.163	607.215± 131.988	14.721	0.000*
		right	402.551 ± 156.515^c,d^	405.1833± 182.597^c,d^	516.582± 173.001^d^	621.162± 134.222	15.352	0.015*
the ratio of pressure onset time#	the heel	left	0.000 (0.500)	0.000 (0.500)	0.000 (0.000)	0.000 (0.000)	3.804	0.283
		right	0.000 (0.500)	0.000 (1.630)	0.000 (0.500)	0.000 (0.500)	1.149	0.765
	middle foot	left	3.000 (4.750)^d^	3.000 (3.130)^d^	2.250 (5.130)^d^	5.500 (3.500)	18.433	0.005*
		right	2.500 (4.000)^d^	3.250 (4.130)^d^	3.250 (5.000)^d^	6.500 (3.630)	26.525	0.016*
	anterior foot	left	1.000 (3.500)^d^	1.500 (4.000)^d^	1.500 (4.250)^d^	5.250 (3.130)	35.717	0.000*
		right	1.500 (2.750)^d^	2.500 (4.130)^d^	2.500 (4.630)^d^	6.000 (2.630)	39.083	0.000*
TA	Standing RMS(μV)#	Left	85.700 (85.400)	91.450 (96.930)	110.500 (124.600)	110.00 (64.700)	4.608	0.203
		right	86.700 (77.050)	69.800 (74.230)	83.450 (102.330)	82.950 (57.650)	2.808	0.422
	Swing RMS(μV)#	Left	49.700 (49.550)	62.700 (40.530)	64.050 (61.830)	65.700 (34.100)	6.259	0.100
		right	52.300 (36.600)	44.350 (41.480)^d^	50.600 (36.250)	53.350 (38.450)	8.805	0.028*
	MPF	Left	92.756 ± 22.464	94.117 ± 23.232	96.776 ± 27.371	102.794 ± 24.577	1.240	0.297
		right	71.104 ± 18.263	73.141 ± 21.201	75.679 ± 23.979	100.779 ± 25.161	0.933	0.427
	MF	Left	91.578 ± 24.165	98.874 ± 21.927	96.474 ± 21.817	78.932 ± 23.694	1.198	0.312
		right	71.496 ± 21.368	76.564 ± 17.217	74.458 ± 18.752	80.671 ± 22.140	1.446	0.232
GM	Standing RMS(μV)#	Left	93.900 (153.250)^d^	111.000 (154.450)	128.000 (151.500)	173.000 (301.730)	10.653	0.009*
		right	111.000 (102.250)	87.700 (84.400)^d^	111.500 (115.270)	154.000 (159.880)	10.698	0.011*
	Swing RMS(μV)#	Left	33.900 (53.400)^d^	35.800 (66.250)^d^	47.050 (70.130)	68.250 (208.380)	10.761	0.049*
		right	36.100 (63.100)	26.850 (29.580)	37.900 (62.470)	44.350 (175.980)	5.971	0.113
	MPF	Left	97.444 ± 25.011	97.945 ± 23.435	95.255 ± 33.547	92.759 ± 29.647	0.270	0.847
		right	76.749 ± 22.438	79.074 ± 22.911	76.029 ± 31.497	101.782 ± 29.623	0.201	0.896
	MF	Left	95.861 ± 27.262	103.615 ± 26.667	98.903 ± 25.862	74.468 ± 9.415	0.658	0.579
		right	75.329 ± 28.855	82.835 ± 24.645	78.250 ± 23.039	80.606 ± 26.500	0.659	0.579

Note: #:The skewed distribution data are reported as the median and interquartile distances.

RMS: root mean square, TA: tibial anterior, GM: gastrocnemius medialis. MPF: average power frequency; MF: median frequency.

* Indicates P< 0.05, which was considered to indicate statistical significance.

After multiple comparisons using Tukey’s test and Dunn-Bonferroni test.

^a-d^ indicates P< 0.05 when compared to group (a)- (d).

### 3.4 Muscle activation parameters

**FIGURE 3:**
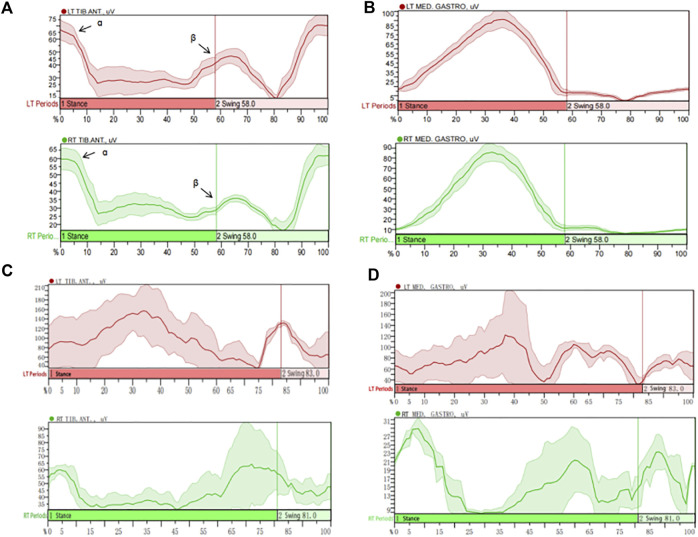
Muscle activation characteristics of the tibialis anterior (TA) and the gastrocnemius medialis (GM) during the gait cycle in healthy elderly individuals and LDG6 elderly individuals. Based on the resulting left and right vertical ground reaction curve each heel strike and toe off are determined *via* mode “Rise/Fall by trigger channel”, “Rise to rise with event” and “Relative” threshold criteria of 1% change (between local min and max value within trigger signal). MATLAB used IMU’s vertical foot accelerometer data to identify heel strikes and extract temporal features of gait. Then trials were parsed into gait cycles using heel strike timings and resampled to data points per gait cycle. **(A)** The activation pattern of the TA in healthy elderly individuals was typical bimodal activation (alpha and beta peaks). **(B)** The activation pattern of the GM in healthy elderly individuals was typical unimodal activation. **(C)** The typical sEMG signal in the TA of LDG6 elderly individuals showed that the RMS curve was not smooth, and the bilateral RMS peak was asymmetric. The activation time of the left TA was delayed, and the peak of the right TA was significantly reduced. **(D)** The typical sEMG signals of in the GM of LDG6 elderly individuals showed that the RMS curve was not smooth, and the bilateral RMS peak was asymmetric. The activation time of the left GM was delayed, and the peak of the right GM was significantly reduced. Note: TIB: tibialis anterior; MED: gastrocnemius medialis.

Compared with those in the healthy control group, the root mean squares (RMS) in the swing phase of right TA, standing phase of bilateral GM and swing phase of left GM were significantly lower in the LDG4, 5, 6 groups (*P* < 0.05). Moreover, there was no significant difference in the average power frequency or median frequency (*P* > 0.05) (Table 5). This suggested that the RMS of the GM was positively correlated with LDG, while the TA showed no regularity. The contraction of the TA and the GM of the lower extremities was coordinated and stable during walking in healthy elderly individuals, and there was no significant difference in the myoelectrical parameters (*P* > 0.05) (Supplementary Table S1). Moreover, the activation pattern of the TA in healthy elderly individuals during the gait cycle was typical bimodal activation (alpha and beta peaks), while the activation pattern of the GM was typical unimodal activation. However, the elderly individuals in the LDG4, 5, 6 groups exhibited abnormal activation patterns. The RMS curve was not smooth, the bilateral RMS was asymmetrical, the activation time of the TA and the GM was delayed, and the peak values were decreased (Figure 3).

## 4 Discussion

At present, there are no clear studies and analysis on gait parameters and sEMG parameters of elderly individuals with different LDG grading. This study proposed to use LDG scale combined with objective detection to evaluate elderly individuals with different LDG grading. Through exploring the differences and regularity of gait and muscle activation characteristic parameters in the LDG scale assessment, analyse the correlation between objective parameters and scale grading. It proposed reference for intelligent evaluation and rehabilitation, and digital medicine.

In this study, gait asymmetry occurred in both the LDG4, LDG5, LDG6 groups and the healthy control group. Compared with those in the healthy control group, elderly individuals in the LDG4, LDG5, LDG6 groups needed minimal or substantial assistance to some extent for basic and instrumental activities of daily living due to impaired mobility. Gait features decreased significantly, including step velocity, cadence, step length and stride length, which was consistent with the findings of Lilian et al. in a community-dwelling elderly individuals in 2021 (Motti et al., 2021). Ageing is accompanied by a decrease in hormone levels and immune capacity and endocrine system function, and the rate of muscle protein breakdown exceeds the rate of synthesis. As a result, the number of muscle fibers in elderly individuals is reduced, and the muscle strength of the lower extremities is weakened (Attwaters and Hughes, 2022), which is ultimately reflected in a decrease in walking ability (Shinohara et al., 2022). A reduced step velocity is considered an important predictor of balance dysfunction (Cruz-Jimenez, 2017). The self-selected velocity of elderly individuals decreased by approximately 18% per 10 years (Grimmer et al., 2019). This may be a response by elderly individuals to maintain balance, which has been strongly associated with motor dysfunction according to numerous studies (Wennie Huang et al., 2010). In this study, with increasing LDG, the differences in spatiotemporal parameters between elderly individuals with lower extremity motor dysfunction and healthy elderly individuals increased in a stepwise manner, indicating that the degree of lower extremity dysfunction became more serious. This predicted a decline in physical function (Gueugnon et al., 2019), muscle weakness, slow reaction movements and loss of walking ability in daily activities (Kitamura et al., 2021). Moreover, the daily ability and independence in activities of elderly individuals are reduced, which seriously affects quality of life (Albert et al., 2015; Nascimento et al., 2022). Therefore, spatiotemporal gait parameters can objectively assess lower extremity motor function in elderly individuals. The standing phase, load response, double support phase, step width and walking time were positively correlated with the LDG, while the swing phase, step length, stride length, velocity and cadence were negatively correlated.

In the gait cycle, elderly individuals with motor dysfunction need to lengthen the support phase time to maintain balance and ensure the steady progression of the centre of gravity. Therefore, the support time for both legs is significantly longer in elderly individuals than in healthy elderly individuals, resulting in a significant decrease in the proportion of bilateral swing phase (Laufer, 2005). The increased double support phase is intended to compensate for balance and stability of the body, avoiding falling and successfully completing the initiated gait. The increased support phase may also be an important marker of age-related movement changes, indicating impaired postural control during gait in elderly individuals. This study suggested that the walking cycle of the LDG4, 5, 6 groups was significantly longer than that of the healthy elderly group. The proportion of individuals in the support phase increased throughout the whole walking cycle, and the proportion of individuals in the swing phase decreased (Michalska et al., 2021). Elderly individuals with lower extremity mobility dysfunction have limited swing amplitude and frequency in the lower extremities and reduced ability to control movement while walking. By reducing the proportion of swing phase and increasing the proportion of support phase, this compensatory walking mode may better maintain body balance and thus reduce the risk of falling (Park et al., 2018; Sittichoke et al., 2019).

The changes in gait parameters in elderly individuals included not only spatiotemporal parameters but also movement parameters. A normal gait is affected by the hip joint, knee joint and ankle joint. In this study, compared with those in healthy elderly individuals, bilateral hip flexion, hip abduction, knee flexion and ankle dorsiflexion angles in the LDG4, 5, 6 groups were significantly lower. These findings indicated that the joint motion angle was negatively correlated with the LDG. With increasing age and knee and ankle joint disease severity, the physiological structure inside the knee and ankle joint of the human lower extremity will change, which will continuously affect the mechanical structure of the joint. However, motor and sensory functions decline, leading to changes in the functional trajectory of motor performance. From the perspective of kinematic characteristics, the walking characteristics of elderly individuals include a reduction in the hip joint extension angle, ankle joint dorsiflexion and plantar flexion angle (Kerrigan et al., 1998; DeVita and Hortobagyi, 2000; Calderón and Ulloa, 2016). These differences may be associated with actual gait-limiting factors and neuromuscular adaptation with aging, or simply a conscious choice of movement patterns to produce a slower gait. Age also causes a redistribution of torque and force in the joints. When walking at the same speed, elderly individuals use their hip extensors more than younger individuals do, and their knee extensors and ankle plantar flexors less. Consistent with these results, compared with the healthy control group, elderly individuals in the LDG6 group had a significantly smaller knee flexion angle during the swing phase. Compared with those of the hip joint and ankle joint, the knee joint flexion angle in elderly individuals was more varied during the swing phase. Knee joint flexion is used to prepare for foot clearance caused by the foot pushing off the ground. When the heel is off the ground and the toe is off the ground, the lower extremity is driven by knee flexion.

The 95% confidence ellipse was calculated as a reliable method for assessing postural stability. The results suggested that the 95% confidence ellipse was significantly greater for elderly individuals with motor dysfunction than for healthy elderly individuals. To maintain the stability of the body posture, elderly individuals can achieve stable movement at the centre of gravity and control of posture by expanding the area of the ellipse with a large swing. As one of the parameters of gait kinematics, the COP is an effective index for assessing postural stability. The COP path length refers to the total length of the COP moving in a certain period of time and is the sum of the point spacings of adjacent COPs. When conducting large-scale balance measurements, this index is accurate and effective. The smaller the value is, the better the postural stability. The results of this study showed that, compared with that of the healthy control group, the COP path length of LDG4, 5, 6 groups was significantly longer, showing a gradual upward trend. The more severe the degree of motor dysfunction is, the longer the COP path length and the worse the stability. The average COP velocity exhibited the opposite trend. The 95% confidence ellipse and COP path length were positively correlated with the LDG, while the COP average velocity was negatively correlated. These gait kinematic parameters showed strong reliability and clinical practicability.

The walking process of humans involves fine and complex nerve regulation. Different muscles contract in a coordinated and orderly manner under the innervation of nerves to complete various functional actions. As an important part of clinical gait analysis, sEMG has been proven to be closely related to muscle function status, and the working characteristics and regularity of muscles during movement can be obtained (Papagiannis et al., 2019; Xiong et al., 2020). With increasing age, elderly individuals will experience a series of reactions, such as decreased muscle strength, increased muscle reaction time and fear of falling. sEMG is often placed in the TA, gastrocnemius lateralis (GL) and GM in the study of lower extremity motor function in elderly individuals (Marques et al., 2022). Joint contraction of the tibialis anterior muscle and the gastrocnemius muscle is used as a compensatory strategy to enhance stability, and maintaining balance around the ankle becomes an ankle joint strategy. Therefore, the TA and the GM were selected as the main muscles to evaluate the walking process of lower extremities in the elderly individuals. However, the thigh muscle were not included in this study. Mobarak (Mobarak et al., 2024) proposed that EMG data from the thigh could carry important neuromuscular information regarding the evolution of human gait, suggesting the importance of thigh muscle. We have considered it, but when evaluating the gait and sEMG data, the sensors at the bilateral knee joints can conflict with the surface electrodes of the thigh muscles, interfering with the accuracy of the data. It is also one of the key technical issues we need to overcome in the future study.

In healthy elderly individuals, the TA and the GM muscle of both lower extremities were activated and coordinated during walking. The TA muscle showed a typical bimodal activation pattern during the gait cycle, with the first activation peak (alpha peak) occurring in the load response phase of the standing phase and the second activation peak (beta peak) occurring in the preswing phase. The GM exhibited a typical unimodal activation pattern during the gait cycle. Its peak activation occurs at the end of standing, when it contracts to ensure that body’s centre of gravity shifts (Li et al., 2020). In this study, sEMG analysis of the LDG4, 5, 6 groups showed significant changes in muscle activation in elderly individuals with functional dysfunction. The sEMG data from the standing phase and the swing phase showed that muscle control in the TA and the GM muscle was impaired, and the activation time of the muscles was delayed. The RMS of the GM was positively correlated with LDG, while the TA showed no clear correlation. Moreover, there was a tendency for overlapping activation between the two muscles. The bilateral RMS values were asymmetrical and lower on one side. This pair of antagonistic muscles exhibited a co-contraction phenomenon, an ineffective muscle coordination strategy that can cause joint stiffness or postural abnormalities (Lo et al., 2017) and significantly increase energy expenditure during movement. Elderly individuals may unconsciously use co-contraction to cause joint stiffness to compensate for the deterioration of postural control and sensory processing. Therefore, it may be important to reduce lower extremity co-contraction in elderly individuals to improve gait biomechanics and balance and reduce mobility impairment and the risk of falls.

## 5 Conclusion

Through simultaneous analysis of sEMG and gait, this study explored the gait and muscle activation characteristics of elderly individuals in the LDG4, 5, 6 groups and revealed a deterioration in walking stability and bilateral gait asymmetry. With increasing LDG, the differences in spatiotemporal, kinematic and dynamic gait parameters between elderly individuals with motor dysfunction and normal individuals gradually increased. The sEMG parameters showed an abnormal activation pattern. The first combination of gait and sEMG with LDG scale can provide a more comprehensive and objective assessment of lower extremity motor function in elderly individuals, improve assessment accuracy, and help accurate rehabilitation. At the same time, the dual data of scale evaluation and objective detection provides evidence for intelligent evaluation and rehabilitation, and digital medicine. Moreover, this approach also provides an objective basis for the classification of care levels in elderly care policies.

## 6 Limitations

This study has several limitations. The wearable Noraxon gait analysis system used in this study required high-speed cameras to synchronize with the sEMG device. This limited the ability of a more comprehensive summary of gait analysis and muscle activation regularity in elderly individuals. At the same time, although the sensor and electrode shedding caused 3% data loss, it did not affect the study results. Moreover, the existing motion measurement and quantitative analysis methods cannot fully meet clinical application requirements. However, there are still technical difficulties in the measurement of gait kinematic parameters and the extraction of gait features. How to extract highly sensitive characteristic indicators to help judge lower extremity motor function in elderly individuals and realize multisource data fusion are still problems that need continuous attention in clinical research.

## Data Availability

The original contributions presented in the study are included in the article/Supplementary Material, further inquiries can be directed to the corresponding author.

## Abbreviations

sEMG, surface electromyography; LDG, Locomotion Dysfunction Grade; IMUs, inertial measurement units; ADL, activities of daily living; TA, tibialis anterior; GM, gastrocnemius medialis; SENIAM, Surface Electromyography for the Non-Invasive Assessment of Muscles; ISB, International Society of Biomechanics; TIB, tibialis anterior; MED, gastrocnemius medialis; GL, gastrocnemius lateralis; COP, centre of pressure; RMS, root mean square; MPF, average power frequency; MF, median frequency.
