# Supplementary material for: The correlation of gait and muscle activation characteristics with locomotion dysfunction grade in elderly individuals
Source: Front Bioeng Biotechnol. 2024 Aug 5;12:1372757. doi: 10.3389/fbioe.2024.1372757 (PMC11331308; doi:10.3389/fbioe.2024.1372757)
Supplement: Supplementary file 1 [file DataSheet1.docx]

Supplementary Material

# Supplementary Figures and Tables

## Supplementary Figures


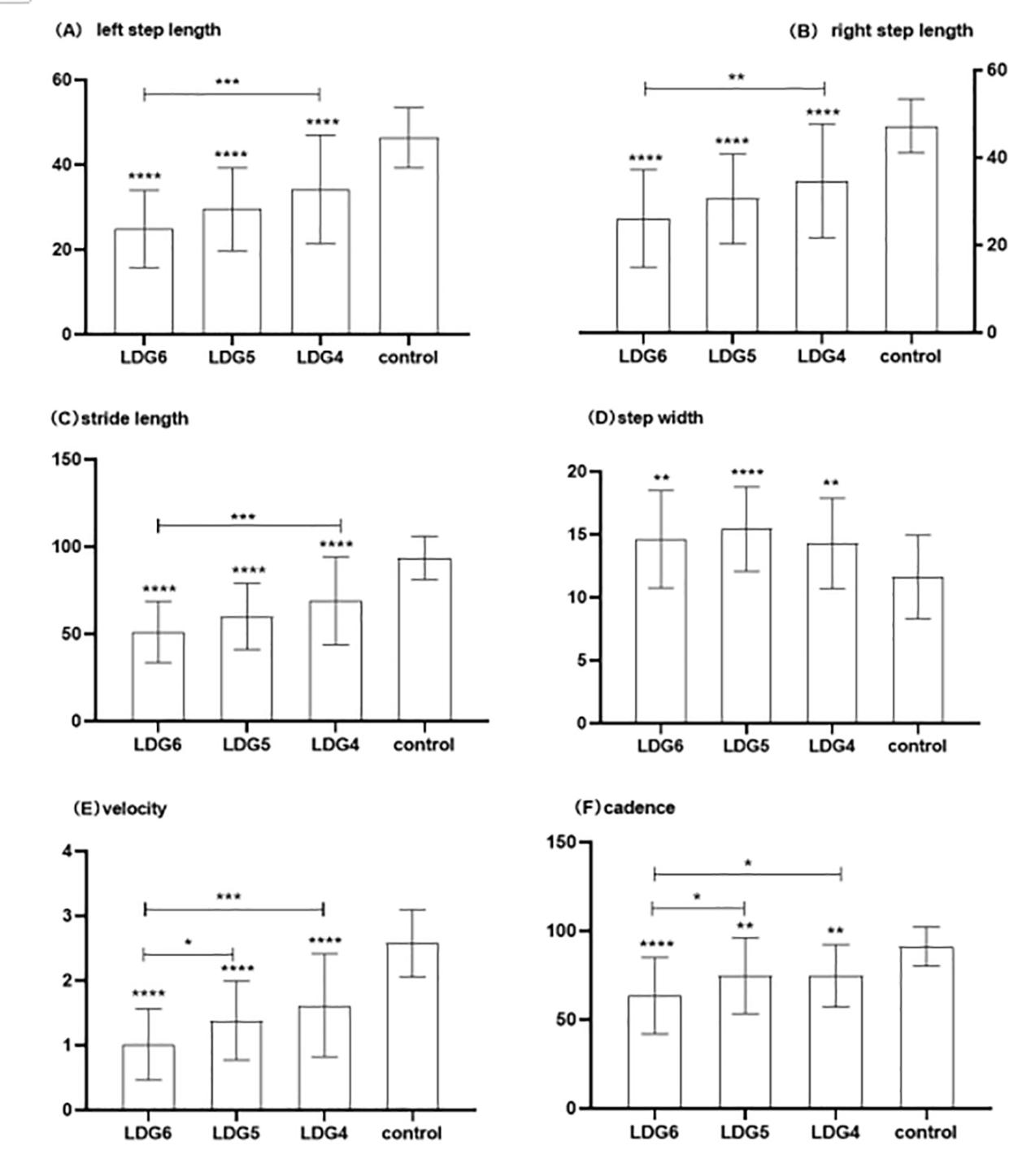


**Supplementary Figure 1.** Comparison of bilateral step length, stride length, step width, velocity and cadence between the LDG4, 5, 6 groups and healthy control group.

A: left step length, B: right step length, C: stride length., D: step width, E:velocity, F:cadence .


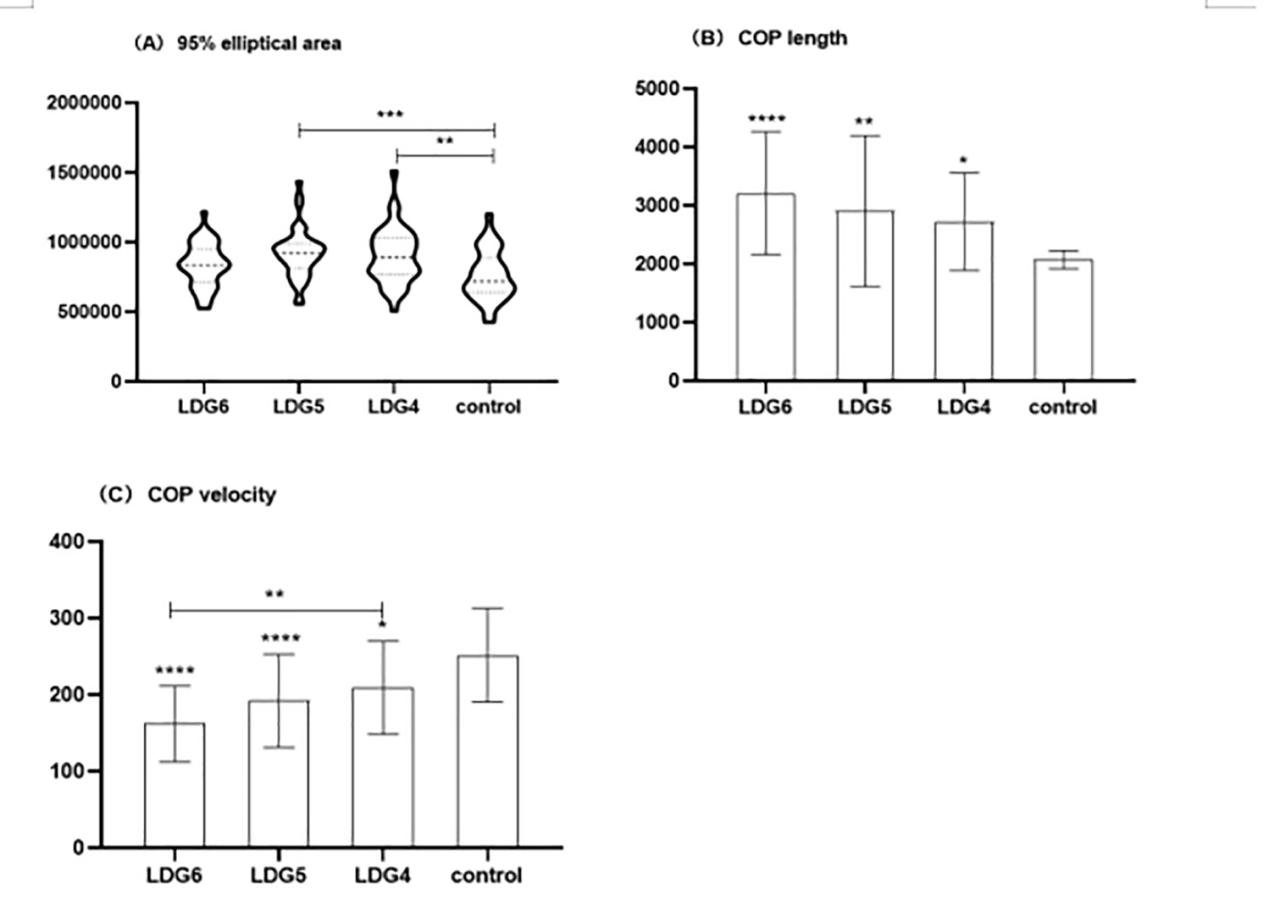


**Supplementary Figure 2.** Comparison of gait kinematic COP parameters between the LDG4, 5, 6 groups and healthy control group.

A: 95% confidence ellipse, B: COP path length, C: average COP velocity.


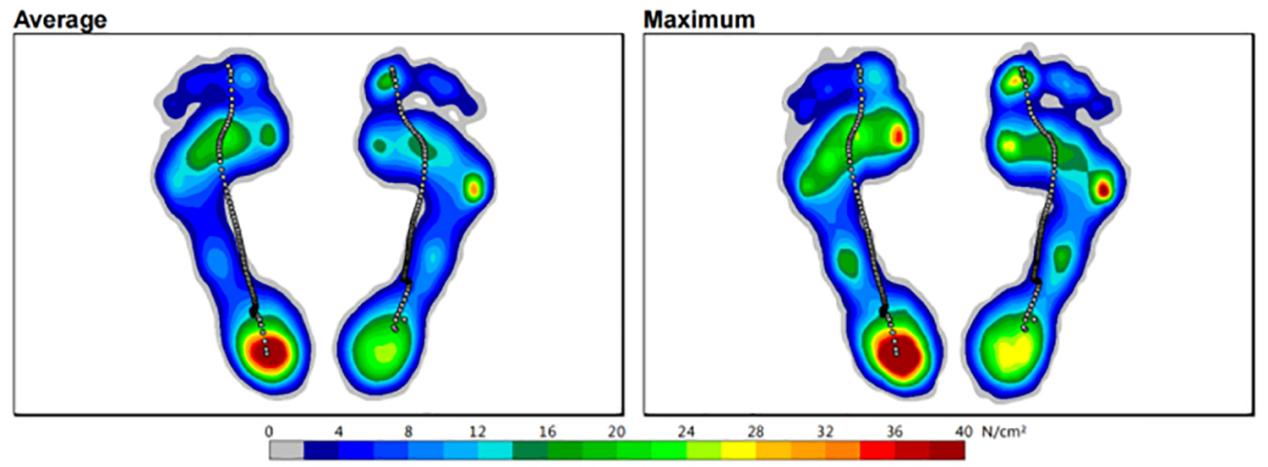


**Supplementary Figure 3.** Typical plantar pressure distribution in elderly individuals in the LDG6 group

The pressure distribution at the bottom of the feet is asymmetrical. When walking, the pressure centre is slightly to the left and the transfer is unstable.The trend of the COP is abnormal and unstable.

## Supplementary Tables

**Supplementary Table 1.** muscle activation characteristics of healthy elderly individuals

|  |  | **left** | **right** | **X^2^/F** | **P** |
| --- | --- | --- | --- | --- | --- |
| TA | Standing RMS（μV） | 144.779±189.794 | 112.735±102.631 | 0.511 | 0.477 |
|  | Swing RMS（μV） | 72.485±35.432 | 82.829±87.162 | 3.780 | 0.056 |
|  | MPF | 102.794±24.577 | 100.779±25.161 | 1.240 | 0.718 |
|  | MF | 78.932±23.694 | 80.671±22.140 | 0.016 | 0.899 |
| GM | Standing RMS（μV） | 577.915±1121.699 | 344.665±891.499 | 2.108 | 0.151 |
|  | Swing RMS（μV） | 329.026±709.494 | 210.101±613.138 | 1.206 | 0.276 |
|  | MPF | 92.759±29.647 | 101.782±29.623 | 0.020 | 0.887 |
|  | MF | 74.468±29.415 | 80.606±26.500 | 0.117 | 0.733 |

Note:#：The skewed distribution data are reported as the median and interquartile distances.

RMS: root mean square, TA: tibial anterior, GM: gastrocnemius medialis. MPF: average power frequency; MF: median frequency.

* indicates P<0.05, which was considered to indicate statistical significance.

# Appendix

## Data processing methods

After the test, the gait and sEMG data can be automatically imported into MATLAB software for data processing and analysis. The original sEMG data was processed by full wave rectification, smooth filtering and RMS processing. In the processing of RMS, the window constant was set to 200ms. All RMS value in each gait cycle were calculated respectively, and the average RMS was obtained after normalization processing. Then the distribution diagram of RMS in the gait cycle was obtained, so as to get the average standing RMS and swinging RMS. After noise reduction, mean power frequency and median frequency, as well as gait spatio-temporal, kinematics and dynamics parameters were automatically imported into MATLAB software. The frequency domain parameters and gait parameters of each gait period were obtained by calculation. The specific operation interface can be seen in Supplementary Figure 4.


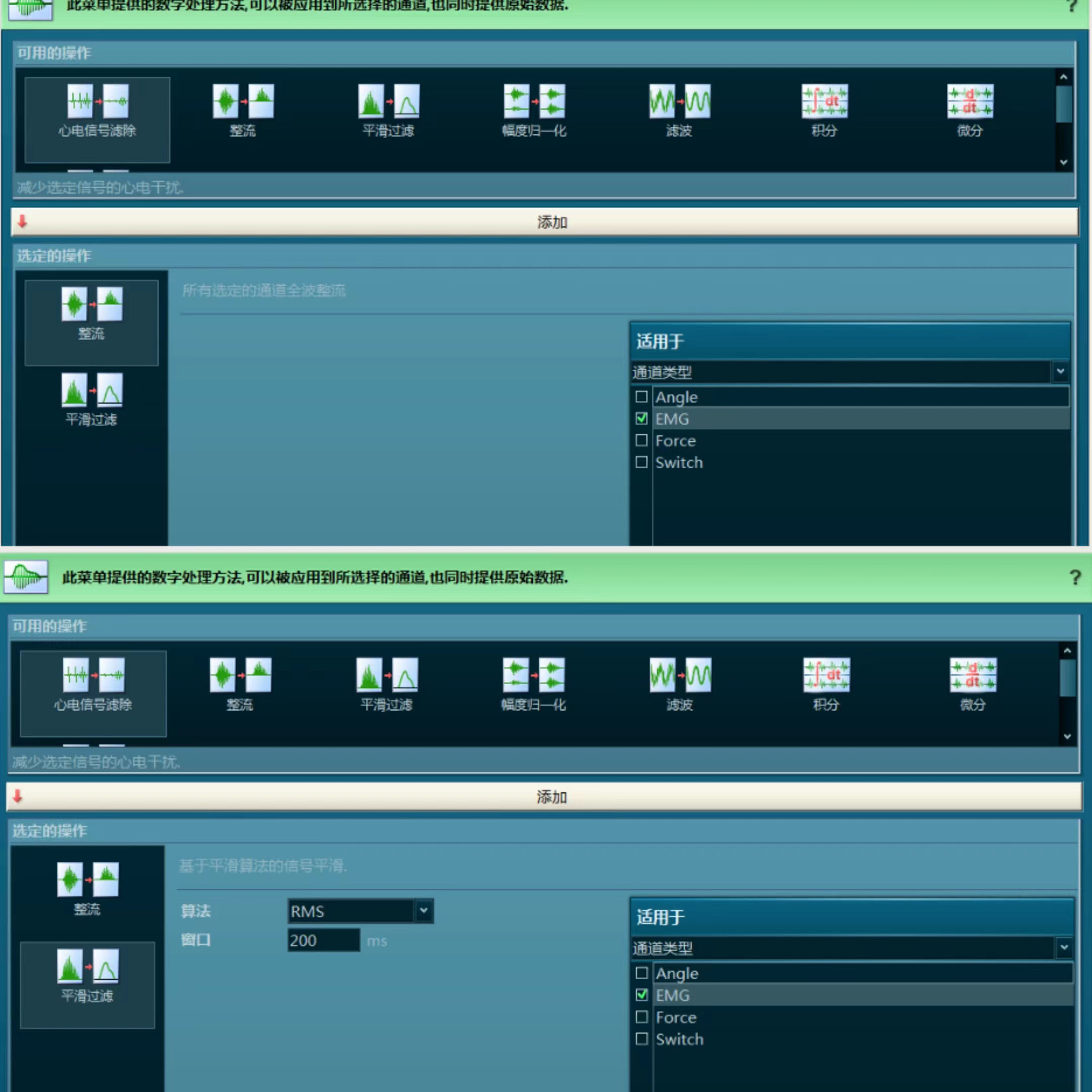


**Supplementary Figure 4.** Data processing operation interface
